# Verbal Learning and Hemispheric Asymmetry

**DOI:** 10.3389/fpsyg.2021.809192

**Published:** 2022-01-04

**Authors:** Vanja Kljajevic

**Affiliations:** Department of Neuromedicine and Movement Science, Faculty of Medicine and Health Sciences, Norwegian University of Science and Technology (NTNU), Trondheim, Norway

**Keywords:** verbal memory, hemispheric asymmetry, arcuate fasciculus (AF), cognitive sex differences, planum temporale (PT)

Cognitive sex differences have been the focus of much research in psychology since the 1960s. The idea that women outperform men in some aspects of verbal learning (e.g., Bleecker et al., 1988; Kramer et al., 1997; Ragland et al., 2000; Sundermann et al., 2016), whereas men outperform women in math and spatial reasoning (e.g., Meinz and Salthouse, 1998; Maguire et al., 1999) has been a matter of debate, in which an opposing stance is formulated as the gender similarity hypothesis (Hyde, 2005, 2016). According to this hypothesis, men and women perform at comparable levels in most cognitive tasks, with major differences arising in their motor abilities, sexual behavior, and aggression.

A recent meta-analysis of 617 studies involving 1,233,921 participants suggests an overall female advantage for episodic memory, including verbal tasks at the levels of word, sentence and discourse, retrieval of names for images and locations, as well as recognition of faces, odors, colors, and tastes, and a male advantage in spatial tasks, including remembering of abstract images and routes (Asperholm et al., 2019a). The observed sex differences were smaller in childhood and old age than in other ages. The female advantage in verbal and other episodic memory tests, which was observed in a sample that was followed for over 40 years, has been related to the fact that, overall, women benefit more than men from societal improvements, such as better education, employment opportunities, and cognitively more stimulating environments, because women might have been at a more disadvantageous level at start (Asperholm et al., 2019b). This view is supported by the findings from a large scale study with 34,300 individuals between 50 and 84 years of age that were recruited across Europe: the Flynn effect, i.e., improvement over time in cognitive performance in people of the same age in new generations, was greater in women than in men for both episodic and semantic memory, although the magnitude of the effect varied across regions (Weber et al., 2017).

Apart from societal factors, approaches attempting to explain sex differences in verbal learning have focused on brain anatomy as well as on factors such as sex hormones. That this is a more complex issue than what it may appear to be at first glance suggest the findings indicating that a sexual dimorphism in adult regional brain volume can be completely reversed by hormonal manipulation (Cooke et al., 1999). McCarthy and Konkle (2005) recently proposed a distinction between stable sex differences, i.e., those that “fundamentally and permanently” differ males and females, from a “hormonally modulated response,” that is, a parameter that “varies in meaningful ways in response to changes in adult circulating hormones” (p. 85). They argue against the tendency to equate sexual dimorphism, as found in the differences in reproductive physiology and behavior between males and females, with cognitive sex differences, because that would misrepresent the magnitude of differences in two cases.

Furthermore, cognitive sex differences often imply brain sex differences in the areas that mediate the cognitive function(s) of interest. However, the underlying neural mechanisms of cognitive sex differences are not well-understood and there is currently no consensus regarding whether they are best addressed by focusing on cell and molecular level analyses in animal models, on human behavior, or at some other level (McCarthy, 2015), which leaves the question of how and why sex differences arise in cognition without a straightforward answer.

One neural mechanism presumably giving rise to cognitive sex differences is sex differences in brain hemispheric asymmetry (Levy, 1974, 1978). Asymmetries in organization of brain hemispheres are associated with directional biases known as lateralization, which is one of the basic organizing principles of human cognition. In the 1970s, Levy argued that male brains had more asymmetry than female brains, with the left hemisphere being functionally specialized for verbal processing and the right hemisphere for spatial processing. However, a recent meta-analysis based on findings from 37 studies that involved a total of 2,623 participants suggests that there is “little empirical support” for the hypothesis that cognitive sex differences, including the difference in verbal memory, depend on sex differences in hemispheric asymmetry (Hirnstein et al., 2019). This meta-analysis included nine studies of verbal abilities, among which one study investigated verbal learning (Ragland et al., 2000), another study investigated both verbal learning and verbal fluency (Catani et al., 2007), whereas the remaining studies investigated verbal fluency or some other verbal ability (e.g., reading skills).

While verbal fluency and word list learning are often used to assess verbal memory, an important difference between the two types of tests is that the former measure vocabulary and semantic memory, whereas the latter measure episodic recall (Andreano and Cahill, 2009). They differ in cognitive complexity and implicate different cognitive processes. Verbal fluency requires access to the mental lexicon prompted by either a semantic (e.g., animal) or a phonological cue (e.g., letter F) and retrieval of all and only those words that fit the search criterion, whereas word list learning involves at least three learning trials of the target list of words, immediate recall of an interference list of words, short and long delayed recall and lastly delayed recognition of the target list. It is therefore not surprising to find different patterns of sex differences in performance in these two types of tests. Catani et al.'s (2007) study, which includes both types of tests, best illustrates the importance of not conflating the processes implicated in these tasks. In that study, women were better than men in verbal learning, but not in verbal fluency. Could sex differences in verbal learning be explained in terms of sex differences in hemispheric asymmetry?

Numerous findings indicate a leftward asymmetry in planum temporale in both sexes, with some evidence suggesting that this asymmetry is significantly more pronounced in male than in female brains, and that other areas in the vicinity of planum temporale, or partly overlapping with it, such as the anterior portions of the middle and superior temporal gyri, show the same pattern: more leftward asymmetry in male brains. There is also evidence on larger right hemisphere planum temporale in female than in male human brains (Ruigrok et al., 2014). However, findings on sex differences in planum temporale asymmetry have been disparate (e.g., Good et al., 2001; Sommer et al., 2008), perhaps due to methodological differences among studies (Buschsbaum et al., 2005). A recent study that included 2,337 individuals from general population, and used relatively uniform methodology, found that the planum temporale showed “the strongest sexually dimorphic asymmetry of any cortical region,” which remained significant after applying a conservative correction for multiple comparisons (Guadalupe et al., 2015, p. 49). The finding on a larger asymmetry in planum temporale in men than in women was corroborated by two other large datasets. These findings are important when assessing verbal learning based on spoken stimuli, because of the critical role of planum temporale in mapping of acoustic speech signals to articulatory frontal lobe networks (Hickok and Poeppel, 2007), as well as because of its role in auditory attention (Hirnstein et al., 2013).

Additionally, asymmetries in white matter connectivity underlying the language network also appear to be relevant for verbal learning. For instance, Catani et al. (2007) found that men had a stronger left hemisphere asymmetry than women in the direct segment of the arcuate fasciculus, and that women performed slightly better than men on the California Verbal Learning Test (CVLT). The arcuate fasciculus is one of the dorsal white matter tracts for language, which connects superior temporal gyrus and inferior frontal gyrus (Kljajevic, 2014). In the tripartite model of the arcuate fasciculus (Catani et al., 2005) that was used in Catani et al.'s (2007) study, the “direct segment” corresponded to the classical concept of arcuate fasciculus. Importantly, this segment has been implicated in word repetition (Catani et al., 2005), and repeating words either out loud or covertly rehearsing them (in one's mind) is an effective strategy to learn new words or lists of words. While women are generally found to have less hemispheric asymmetry than men, which has at times been highlighted as an important factor in men's generally poorer verbal abilities (Levy, 1972, 1976, 1978), Catani et al. (2007) emphasize that it is not the sex, but rather symmetry in the anatomical language pathways that determines better performance in word learning tasks. The idea underlying this argument is that a more symmetric pattern of anatomical connections is associated with a better use of semantic associations (and hence better memory of words) than the extremely left lateralized pattern.

Other biological influences, such as sex hormones, have also been proposed as a plausible explanation of female advantage in verbal learning. As an example, expression of estradiol receptors has been found in the temporal cortex, which underpins the mental lexicon, and in the hippocampus, which supports not only learning in general but—when it comes to words—also word recognition (Bird and Burgess, 2008), syntactic integration (Meyer et al., 2005), and discourse processing (Duff and Brown-Schmidt, 2012), playing an integrative role. Viewed as a function of estradiol, sex differences in verbal memory would mean that women perform better when estradiol levels are higher, which is a notion that many studies failed to support (e.g., Yonker et al., 2003; Herlitz et al., 2013).

As there are many possible sources of sex differences in the brain (McCarthy, 2015), the factors driving cognitive sex differences, including the sex difference in verbal learning, are likely multifaceted as well (Andreano and Cahill, 2009; Asperholm et al., 2019a). For instance, some studies suggest that the female advantage in word list learning might be due to use of an efficient learning strategy (Kramer et al., 1988) and that women are better in utilizing learning strategies than men. In tests of word list learning, such as CVLT, Rey Auditory Verbal Learning Task (RAVLT), and similar, this means a better use of a semantic strategy, specifically semantic clustering, whereas use of other strategies (e.g., serial clustering, reliance on the order of presented words or on phonemic properties of words) is typically less efficient. Semantic clustering is grouping of words based on semantic associations (e.g., according to a semantic category). A critical role of semantic clustering in learning lists of words and a consistently better performance of female than male participants in such tasks was found even in children across a range of ages (Kramer et al., 1997): since boys outperformed girls in vocabulary knowledge, the female advantage in verbal learning was explained by learning strategy, i.e., semantic clustering, not by language function *per se*.

This brings us back to the idea that a better use of semantic associations in simple word tasks may be driven by more symmetry in the relevant brain regions, making a version of Levy's original hypothesis still plausible. Specifically, we refer to the hypothesis that female advantage in verbal learning might be due to sex differences in planum temporale asymmetry.

Since learning lists of words is not a monolithic task, but instead it requires encoding, retention and organization of learned information, which if successful, will enable successful retrieval and recognition, sex differences in word list learning may arise due to differences in any of these key processes. Consider, for example, the notion that sex differences arise at the encoding stage, i.e., the stage during which the incoming information is being committed to short-term memory (Cowan, 2010). Most studies on word list learning take the first learning trial (T1) to be an indicator of attention. In tests using spoken stimuli, T1 would be an indicator of auditory attention. The findings so far indicate that women outperform men in T1 (e.g., Ragland et al., 2000). Since efficiency in encoding determines all subsequent steps in verbal short-term memory tasks (Barry et al., 2011), a plausible explanation is that this initial advantage then leads to an overall female advantage, assuming that men cannot compensate for the difference over the subsequent learning trials. In other words, less efficient encoding leads to less efficient retention, retrieval and recognition.

Importantly, the planum temporale may feature prominently in all still dominant models of short-term memory as well as speech production (Baddeley, 1986; Buschsbaum and D'Esposito, 2008; Hickok et al., 2009; Cowan, 2010). In word list learning, planum temporale may support perception and covert rehearsal of verbal stimuli. There is evidence that the posterior portion of the planum temporale, known as area Spt (Sylvian-parietal-temporal), supports modality-neutral phonological-articulatory memory, while the superior temporal sulcus/superior temporal gyrus has a more limited role, supporting modality-specific echoic memory (Barry et al., 2011). In fact, the planum temporale could contribute to word lists' learning in multiple ways, from supporting the initial encoding stage *via* its role in auditory attention, to support of covert rehearsal in short-term memory regardless of the modality of input (auditory/visual), or by affording code binding (e.g., acoustic and articulatory codes). If hemispheric symmetry is optimal architecture for word list learning and if men have more asymmetry in planum temporale than women, then it is possible that the sex difference in planum temporale asymmetry drives the female advantage in word list learning.

Thus, although learning lists of words is more efficient when strategies such as semantic clustering are used, and although women use these strategies more than men (Andreano and Cahill, 2009), the possibility remains that an initial difference in encoding that could be due to a sex difference in planum temporale asymmetry might be driving this cognitive sex difference.

## Author Contributions

The author confirms being the sole contributor of this work and has approved it for publication.

## Conflict of Interest

The author declares that the research was conducted in the absence of any commercial or financial relationships that could be construed as a potential conflict of interest.

## Publisher's Note

All claims expressed in this article are solely those of the authors and do not necessarily represent those of their affiliated organizations, or those of the publisher, the editors and the reviewers. Any product that may be evaluated in this article, or claim that may be made by its manufacturer, is not guaranteed or endorsed by the publisher.
